# Discovery and genome sequencing of a new virus related to members of the family *Tymoviridae*, isolated from mosquitoes of the genus *Mansonia* in Brazil

**DOI:** 10.1007/s00705-022-05475-x

**Published:** 2022-06-05

**Authors:** Keissy Karoline Pinheiro Miranda, Glennda Juscely Pereira Galvão, Pedro Arthur da Silva Araújo, Ana Claudia da Silva Ribeiro, Sandro Patroca da Silva, Poliana da Silva Lemos, Lívia Carício Martins, Márcio Roberto Teixeira Nunes, Pedro Fernando da Costa Vasconcelos, Vânia da Costa Ferreira, Fábio Medeiros da Costa, Rosemary Aparecida Roque, Wanderli Pedro Tadei, Ana Cecilia Ribeiro Cruz, Valéria Lima Carvalho

**Affiliations:** 1 Department of Arbovirology and Hemorrhagic Fevers, Pos Graduate Program in Virology, Evandro Chagas Institute, Ananindeua, PA 67030-000 Brazil; 2 Center for Technological Innovations, Evandro Chagas Institute, Ananindeua, PA 67030-000 Brazil; 3 Energia Sustentável do Brasil, Porto Velho, Rondônia 76840-000 Brazil; 4 Oikos Consultoria e Projetos, 76801-260 Porto Velho, Rondônia Brazil; 5 Malaria and Dengue Laboratory, National Institute of Amazonian Research (INPA), Manaus, Amazonas 69067-375 Brazil; 6 Department of Pathology, State University of Pará, Belém, PA 66087-670 Brazil

## Abstract

A new virus, named Mutum virus, related to members of the family *Tymoviridae*, was isolated from mosquitoes (*Mansonia* spp.) in clone C6/36 cells, and its complete genome was sequenced. Its genome is 6494 nt in size with an organization resembling that of tymovirids. The isolated virus is phylogenetically related to two viruses isolated from *Culex* spp. mosquitoes: Ek Balam virus, reported in Mexico, and Culex-originated Tymoviridae-like virus, isolated in China. The results of this study suggest that this virus is a new member of the family *Tymoviridae.*

**Supplementary Information:**

The online version contains supplementary material available at 10.1007/s00705-022-05475-x.

The family *Tymoviridae* is composed of three genera of viruses with positive-sense RNA genomes: *Maculavirus*, *Marafivirus*, and *Tymovirus* [1]. Initially, the family *Tymoviridae* comprised only plant-infecting viruses; however, viruses related to members of this family have been isolated from mosquitoes in recent years, such as Culex-originated Tymoviridae-like virus (CuTLV) and the Ek Balam virus (EkBV) [1–3].

Here, we report genome sequencing of another distinct tymo-like virus, Mutum virus (MUTV), named after the mosquito collection area. MUTV was detected in female *Mansonia* sp. mosquitoes collected in 2018 in the vicinity of the Jirau hydroelectric dams in Nova Mutum Paraná, a rural village in the municipality of Porto Velho in the state of Rondônia, Brazil (Supplementary Fig. S1). MUTV was isolated from four pools of female *Mansonia* mosquitoes in *Aedes albopictus* cells (C6/36) [4]. The viruses were lab coded as BE-AR-855909, BE-AR-855928, BE-AR-855911, and BE-AR-855922.

Four MUTV genomes were sequenced on an Ion Torrent PGM platform (Thermo Fisher Scientific, MA, USA) according to the manufacturer's recommendations. A phylogenetic tree was constructed using the maximum-likelihood (ML) method with 1000 bootstrap replicates [5] in the RaxML v.8.0 program [6].

The sequences of four MUTV isolates have been deposited in the GenBank database with the following accession numbers: MUTV-BE-AR-855909 (MT656586), MUTV-BE-AR-855928 (MT656589), MUTV-BE-AR-855911 (MT656587), and MUTV-BE-AR-855922 (MT656588). The four MUTV isolates showed nucleotide sequence identity ranging from 99.9–100% and amino acid sequence identity of 100%, indicating that they are closely related isolates of the same virus. The MUTV genome is 6,494 nt in size, with 5′ and 3′ non-coding regions of 37 nt and 53 nt, respectively. The MUTV genome has three ORFs. ORF1 is 5,331 nt (nucleotide position 38 to 5,368) in size and encodes a 1,776-aa-long protein with an estimated mass of 201,061 kDa that is involved in virus replication. ORF2 spans 732 nt (nucleotide position 5,400 to 6,131) and codes for a 243-aa-long protein with an estimated mass of 26,134 kDa identified as a viral coat protein, while the 3’-proximal ORF 3 is 285 nt long (nucleotide position 6,182 to 6,466) and codes for a 10,729-kDa protein of unknown function. The total readings and the coverage of the genome reads ranged from 55,312 to 139,811 and from 16.10x to 48.47x, respectively.

Searches of the Interproscan databases revealed conserved protein domains for Vmethyltransf, peptidase, helicase 1, and RdRP in the product of ORF1 and a Tymo_coat motif in the ORF2-encoded protein (Fig. 1). As already indicated, no similarities were found between the protein encoded by ORF3 and currently available proteins in the GenBank/NCBI database.

**Fig. 1:**
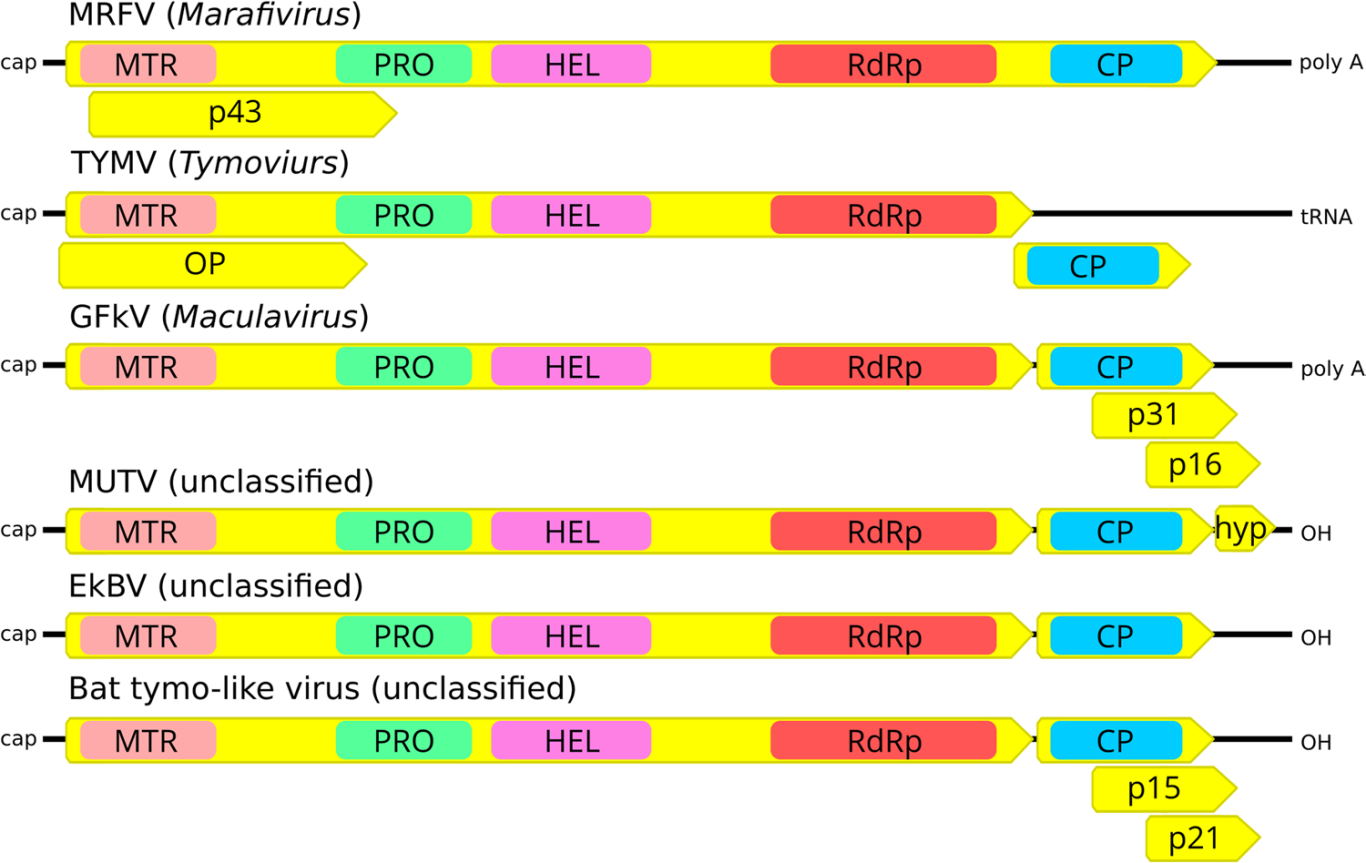
Genome organization of Mutum virus (MUTV) compared to those of members of the three recognized genera in the family *Tymoviridae* and few related but still unclassified viruses. The ORFs are indicated by yellow arrows. The following putative conserved domains are shown in boxes with different colors and were identified using the InterProScan database: MTR, methyltranferase; PRO, papain-like protease; HEL, helicase; RdRp, RNA-dependent RNA polymerase protein; CP, coat protein; OP, movement protein; hyp, hypothetical protein; p43, p31 and p16, proline-rich proteins. MRFV, maize rayado fino vírus; TYMV, turnip yellow mosaic vírus; GFkV, grapevine fleck vírus; EkBV, Ek Balam virus

MUTV is phylogenetically related to two viruses isolated from mosquitoes, EkBV and CuTLV, along with a bat tymo-like virus, forming a clade with bootstrap support of 100%. This clade is most closely related to a group of viruses isolated from bees or from varroa mites, together forming a distinct lineage composed only of insect viruses (bootstrap support, 85%), distinct from the plant-infecting members of the family *Tymovirida*e (Fig. 2). The results of pairwise comparisons show amino acid sequence identity of MUTV with EkBV, CuTLV, and bat tymo-like ranging from 39.0–70.6% and nucleotide sequence identity of 67.0%, 56.3%, and 50.9%, respectively. These values are below the species demarcation threshold for creation of new species in the family *Tymoviridae*.

**Fig. 2:**
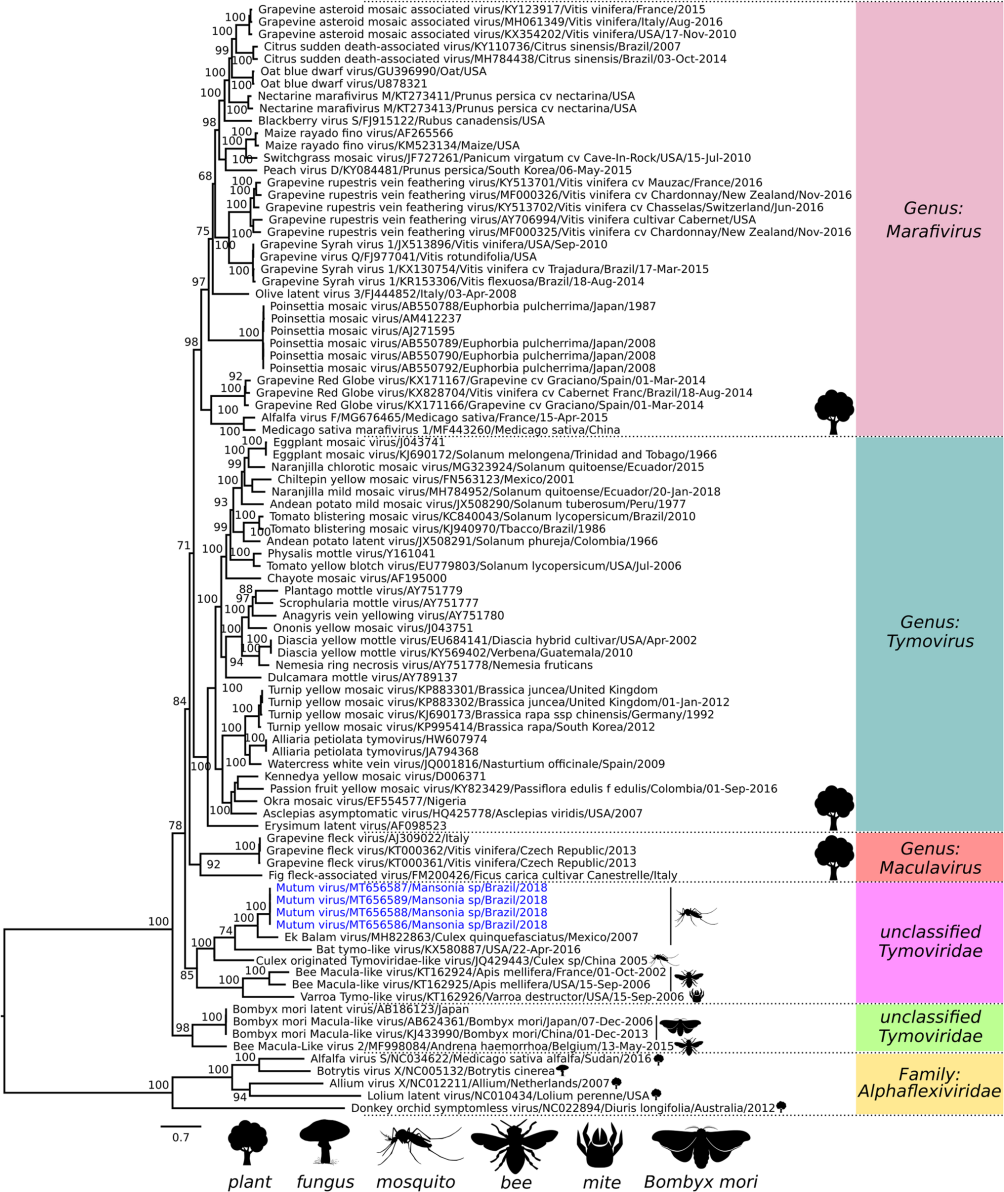
Phylogenetic tree of the family *Tymoviridae* using the ML method based on the complete nucleotide sequences of ORF1. The clusters are labeled in color. The numerical values presented at the nodes of the tree correspond to percent bootstrap values. The scale bar corresponds to genetic divergence. Selected members of the family *Alphaflexiviridae* were used as an outgroup

In summary, here we describe a new mosquito-associated virus named Mutum virus that is related to other tymovirid-like insect specific viruses (ISVs). The viruses isolated from mosquitoes are currently unclassified. The final position of MUTV and other mosquito-infecting tymovirid-like viruses within the ICTV framework of virus classification, in particular within the family *Tymoviridae*, is yet to be determined and may involve establishment of new genera to officially classify these viruses.

## Electronic Supplementary Material

Below is the link to the electronic supplementary material

Supplementary Material 1
