# Supplementary material for: Discovery and genome sequencing of a new virus related to members of the family Tymoviridae, isolated from mosquitoes of the genus Mansonia in Brazil
Source: Arch Virol. 2022 Jun 5;167(9):1889–92. doi: 10.1007/s00705-022-05475-x (PMC9360100; doi:10.1007/s00705-022-05475-x)
Supplement: Supplementary file 1 — Supplementary Material 1 [file 705_2022_5475_MOESM1_ESM.pdf]

Article title: Discovery of a new virus of the family *Tymoviridae* isolated from mosquitoes of the genus *Mansonia* in Brazil

Journal name: Archives of Virology

Author names: Keissy Karoline Pinheiro Miranda[1], Glennda Juscely Pereira Galvão[1], Pedro Arthur da Silva Araújo[1], Ana Claudia da Silva Ribeiro[1], Sandro Patroca da Silva[1], Poliana da Silva Lemos[2], Lívia Carício Martins[1], Márcio Roberto Teixeira Nunes[2], Pedro Fernando da Costa Vasconcelos[6] Vânia da Costa Ferreira[3], Fábio Medeiros da Costa[4], Rosemary Aparecida Roque[5], Wanderli Pedro Tadei[5], Ana Cecilia Ribeiro Cruz[1]*, Valéria Lima Carvalho[1]

Affiliation:

[1] Department of Arbovirology and Hemorrhagic Fevers, and Graduate Program in Virology, Evandro Chagas Institute, Ananindeua, PA 67030-000, Brazil.

[2] Center for Technological Innovations, Evandro Chagas Institute, Ananindeua, PA 67030-000, Brazil.

[3] Energia Sustentável do Brasil, 76840-000, Porto Velho, Rondônia, Brazil.

[4] Oikos Consultoria e Projetos, 76801-260, Porto Velho, Rondônia, Brazil.

[5] Malaria and Dengue Laboratory, National Institute of Amazonian Research (INPA), 69067-375, Manaus, Amazonas, Brazil.

[6] State University of Pará – Department of Pathology, Belém, PA 66087-670, Brazil

E-mail address of the corresponding author: anacecilia@iec.gov.br

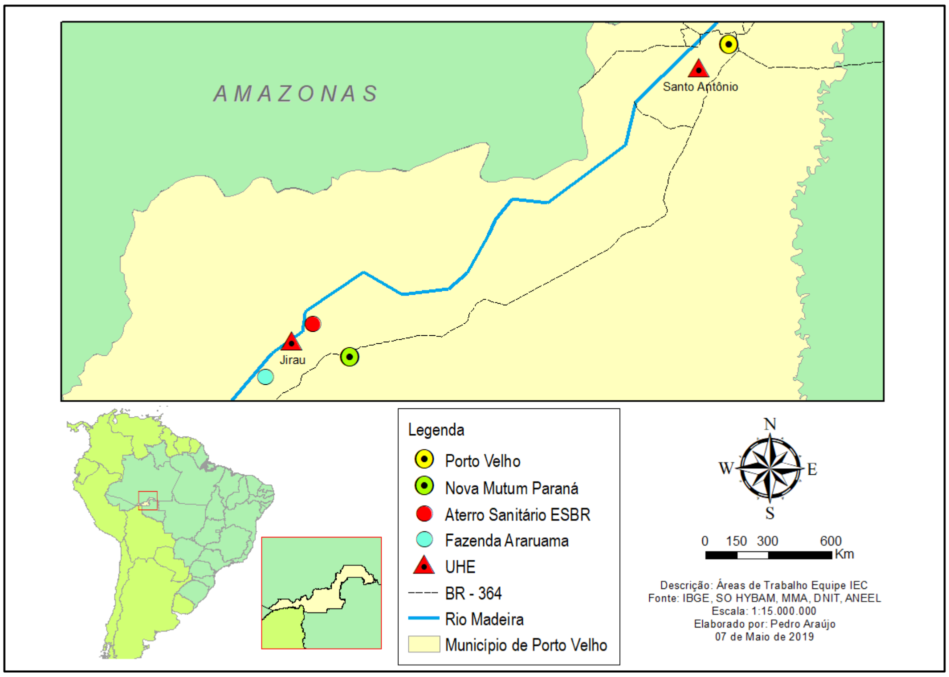

Online Resource 1 - Cartographic representation of the study areas in Nova Mutum Paraná, Rondônia State, Brazil.
